# Multidisciplinary surgical strategy for an invasive thymoma in an immunocompromised patient: a case of a successful resection and postoperative troubleshooting

**DOI:** 10.1186/s44215-025-00192-8

**Published:** 2025-02-03

**Authors:** Tomonari Oki, Shuhei Iizuka, Toru Nakamura

**Affiliations:** Department of Thoracic Surgery, Seirei Hamamatsu General Hospital, 2-12-12, Hamamatsu, Shizuoka, 430-8558 Japan

**Keywords:** Robotic surgical procedure, Transmanubrial osteomuscular sparing approach, Mediastinal neoplasms, Thymoma, Surgical site infection, Mediastinitis

## Abstract

**Background:**

Robot-assisted thoracoscopic surgery has become prevalent as a feasible approach for anterior mediastinal tumor resections, while conventional open surgery, such as a median sternotomy, remains preferred for a combined resection of adjacent organs. However, an additional thoracotomy may be necessary when tumors extend into one hemithorax. This complex approach can cause significant damage to the osseous thoracic cage, increasing the risk of surgical morbidity especially in immunocompromised patients.

**Case presentation:**

A 77-year-old man presented with an anterior mediastinal thymoma measuring 71 mm, detected during an annual health check with suspected involvement of the left brachiocephalic vein and upper lobe of the left lung. The patient had a medical history of recurrent surgical site infections and fasciitis panniculitis syndrome requiring immunosuppressive therapy. To minimize any thoracic cage destruction, a multidisciplinary approach combining robotic surgery with open surgery according to vascular or pulmonary invasion was planned. The patient, initially placed in the supine position with the robot docked over the right side, underwent a thymic dissection, revealing a firm adhesion to the left brachiocephalic vein. The robot was then undocked, and a transmanubrial osteomuscular sparing approach was initiated, enabling a tumor dissection under the proximal and distal control of the left brachiocephalic vein. As invasion into the proximal upper pulmonary vein and extensive dorsal adhesions were observed, the patient was repositioned to the right lateral decubitus position, and a thoracoscopic left upper segmentectomy with adhesiolysis was performed, achieving an R0 resection. The patient was extubated on day 1 but required non-invasive ventilation until day 5. Mediastinitis, likely due to a sternal wire infection, developed on day 9, necessitating debridement, sternal wire removal, and negative pressure wound therapy. After 17 days of treatment, the infection subsided, allowing for a sequestrectomy and chest wall reconstruction with a pedicled pectoralis major myocutaneous flap. By avoiding a total sternotomy, the extent of the mediastinitis was localized, allowing for a limited sequestrectomy. Wound healing was satisfactory, with no recurrent infection at 12 months and minimal functional impairment.

**Conclusions:**

A multidisciplinary approach offers a feasible option for managing an invasive thymoma to minimize postoperative morbidity, particularly in immunocompromised patients. Preoperative surgical planning is essential for guiding intraoperative decision-making and ensuring optimal outcomes.

## Background

In recent years, robot-assisted thoracoscopic surgery has become prevalent as a feasible approach for anterior mediastinal tumor resections due to its superior maneuverability and less invasiveness. Conventional open surgery, such as a median sternotomy, remains preferred in cases requiring a combined resection of adjacent organs. However, an additional thoracotomy may be necessary when tumors extend into one hemithorax. This complex approach can cause significant damage to the osseous thoracic cage, increasing the risk of surgical morbidity. Especially in immunocompromised patients, the risk of postoperative mediastinitis is heightened, complicating subsequent chest wall reconstructions. We report a case of an invasive thymoma in an immunocompromised patient who underwent a curative resection through a multidisciplinary surgical approach, which appeared to minimize the postoperative morbidity, even after the development of postoperative mediastinitis.

## Case presentation

**Fig. 1:**
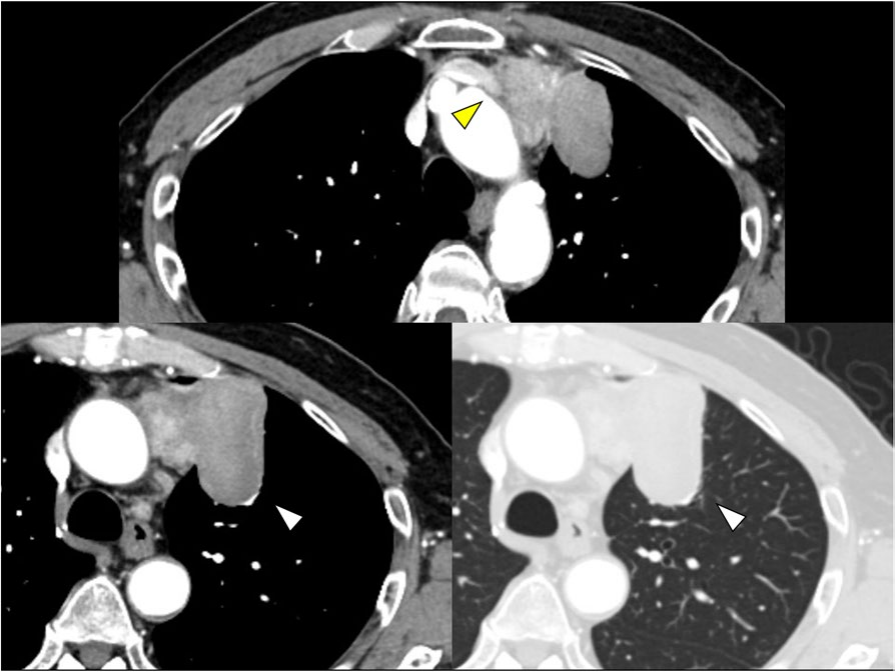
Chest computed tomography revealed a 7.1-cm tumor in the left anterior mediastinum with suspected involvement of the left brachiocephalic vein (yellow arrowhead) and upper lobe of the left lung (white arrowheads)

A 77-year-old man was referred to our hospital for an abnormal mass detected in the mediastinum during his annual medical examination. A chest computed tomography (CT) revealed an anterior mediastinal mass measuring 71 mm in maximum diameter, with suspected involvement of the left brachiocephalic vein and upper lobe of the left lung (Fig. 1). A CT-guided biopsy confirmed a thymoma and a thymothymectomy was planned. The patient did not have any complications associated with thymoma, including myasthenia gravis, but had a medical history of a left shoulder rotator cuff tear treated with a joint replacement, followed by a re-do surgery for a surgical site infection six years prior. He had also developed fasciitis panniculitis syndrome (FPS) that same year and immunosuppressive agents, prednisolone and methotrexate, were initiated. Prednisolone was initiated at a dose of 30 mg/day and gradually tapered to 0.5 mg/day over 4 years. Methotrexate was continuously administered at doses ranging from 8 to 16 mg/week, adjusted based on the clinical symptoms. Four years later, the patient experienced a recurrence of FPS with a reinfection of the left shoulder joint, necessitating another surgery, and the administration of immunosuppressive agents was subsequently discontinued. Postoperatively, a joint contracture developed, requiring 3 months of rehabilitation. A 34 mm anterior mediastinal mass was detected on CT at the time of FPS recurrence two years prior; however, further investigation was not conducted as the patient discontinued follow-up. Given his history of musculoskeletal disorders and recurrent infections, a less invasive approach that would minimize the thoracic cage destruction was preferred. The procedure was initiated with robotic surgery from the right side, with plans to convert to open surgery if there was evidence of invasion into the left brachiocephalic vein and/or the left lung.

**Fig. 2:**
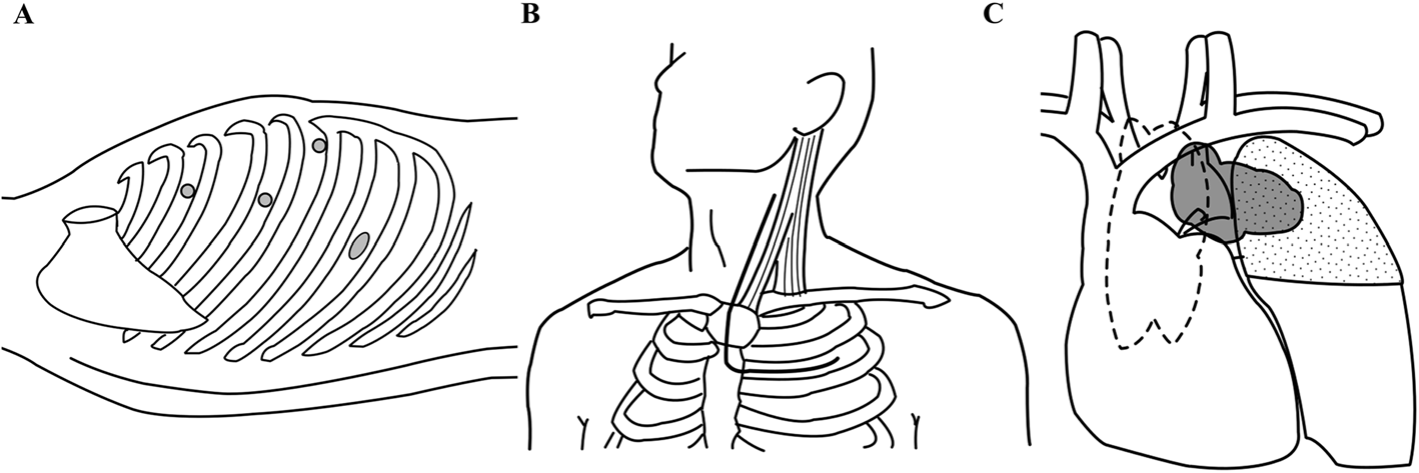
**A** Port placement diagram for robotic surgery. An 8-mm port was placed along the anterior axillary line at the third intercostal space, another 8-mm port at the fifth intercostal space on the same line, an additional 8-mm port ventral to the anterior axillary line at the sixth intercostal space, and a 35-mm assist port was positioned along the mid-axillary line at the eighth intercostal space.** B** Incision for the transmanubrial approach. An L-shaped skin incision was made, extending from the anterior margin of the left sternocleidomastoid muscle, traversing the sternal midline, and following the left second intercostal space. **C** Overview of the surgical procedure. Dissection of the thymic tissue (enclosed by the dashed line) was performed using a robotic approach, followed by tumor dissection (gray area) from the left brachiocephalic vein via the transmanubrial approach. Subsequently, the involved left upper segment (dotted area) was resected en bloc using a thoracoscopic approach

**Fig. 3:**
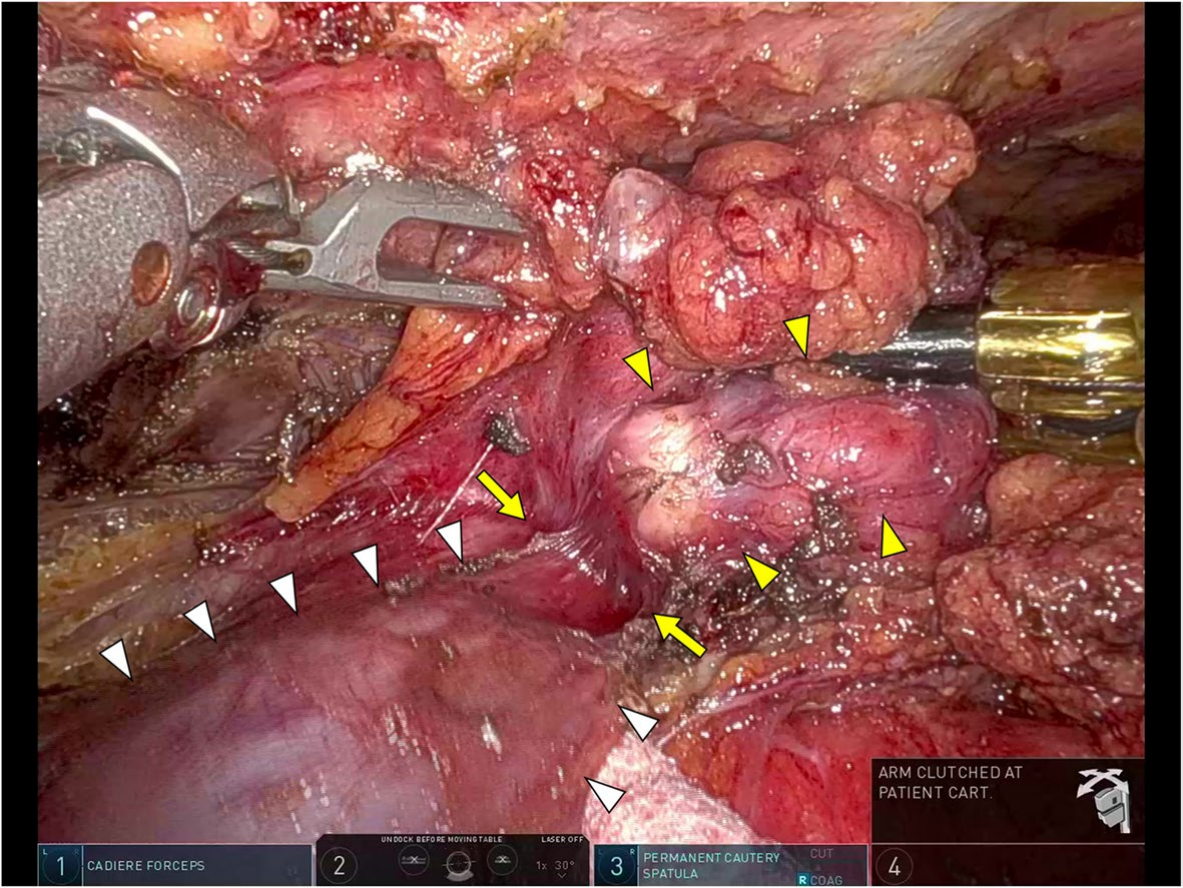
Intraoperative findings of robotic surgery. The tumor (yellow arrowheads) was firmly adhered to the left brachiocephalic vein (white arrowheads). Due to the loss of layers (yellow arrows), it was deemed risky to attempt dissection from the left brachiocephalic vein under robotic surgery

**Fig. 4:**
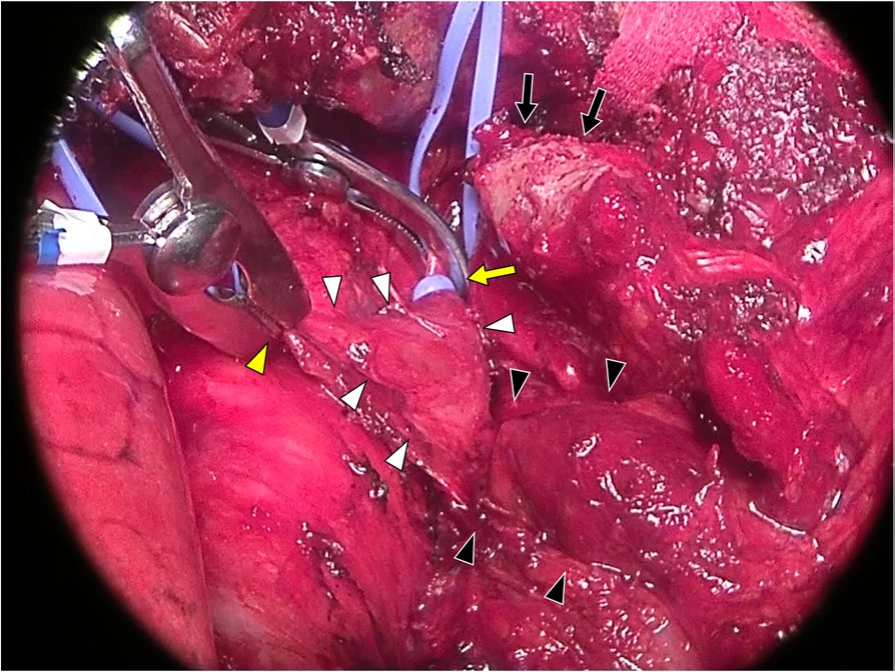
Under the transmanubrial approach, the proximal (yellow arrowhead) and distal (yellow arrow) portions of the left brachiocephalic vein (white arrowheads) were secured, enabling the safe detachment of the tumor (black arrowheads). An optimal field of view was achieved via a transection of the costal cartilage (black arrows)

The patient was placed in the supine position with the robot docked over the right anterior axillary line (Fig. 2A). During the dissection of the thymic tissue, firm adhesion of the mass to the left brachiocephalic vein was noted (Fig. 3). Any lung invasion could not be assessed. The robot was then undocked, and a transmanubrial osteomuscular sparing approach (TMA) was initiated (Fig. 2B). The osteomuscular flap provided optimal exposure of the left venous angle, allowing for precise control of the left brachiocephalic vein. The tumor was dissected successfully under the proximal and distal control of the left brachiocephalic vein, preserving the vein intact (Fig. 4). The TMA also enabled accurate identification of the left vagus nerve at the cervical level, preventing injury to both the vagus and recurrent laryngeal nerves. However, the tumor had invaded the left phrenic nerve, requiring an en-bloc resection of the nerve. Invasion into the proximal upper pulmonary vein indicated the need for anatomical lung resection, and extensive adhesions were observed dorsally. The patient was repositioned to the right lateral decubitus position, and a thoracoscopic left upper segmentectomy with adhesiolysis was performed, achieving an R0 resection (Fig. 2C). The operative times were 179 min for the robotic surgery, 273 min for the TMA, and 208 min for the left upper segmentectomy, with a blood loss of 65 ml and no intraoperative complications.

**Fig. 5:**
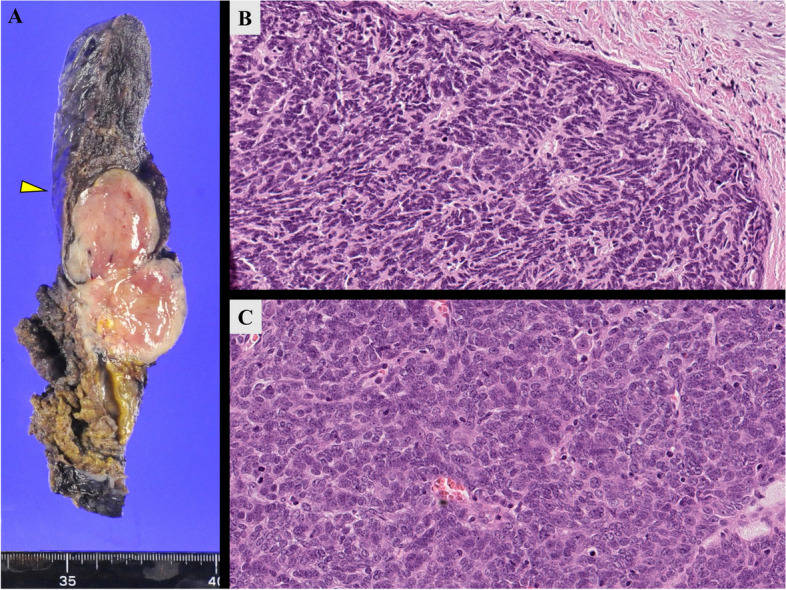
The tumor infiltrated within the left upper lobe of the lung (yellow arrowhead marked in **A**. Hematoxylin–eosin staining (× 20) showed a Type A region (**B**) with spindle-shaped tumor cells proliferating and a Type B region (**C**) with a mixture of small oval cells and lymphocytes

The pathological findings revealed a type AB thymoma measuring 6.2 × 4.8 cm, with invasion into the mediastinal adipose tissue and the lung parenchyma without invasion into the pulmonary vein, consistent with T3N0M0 stage IIIa (Fig. 5).

**Fig. 6:**
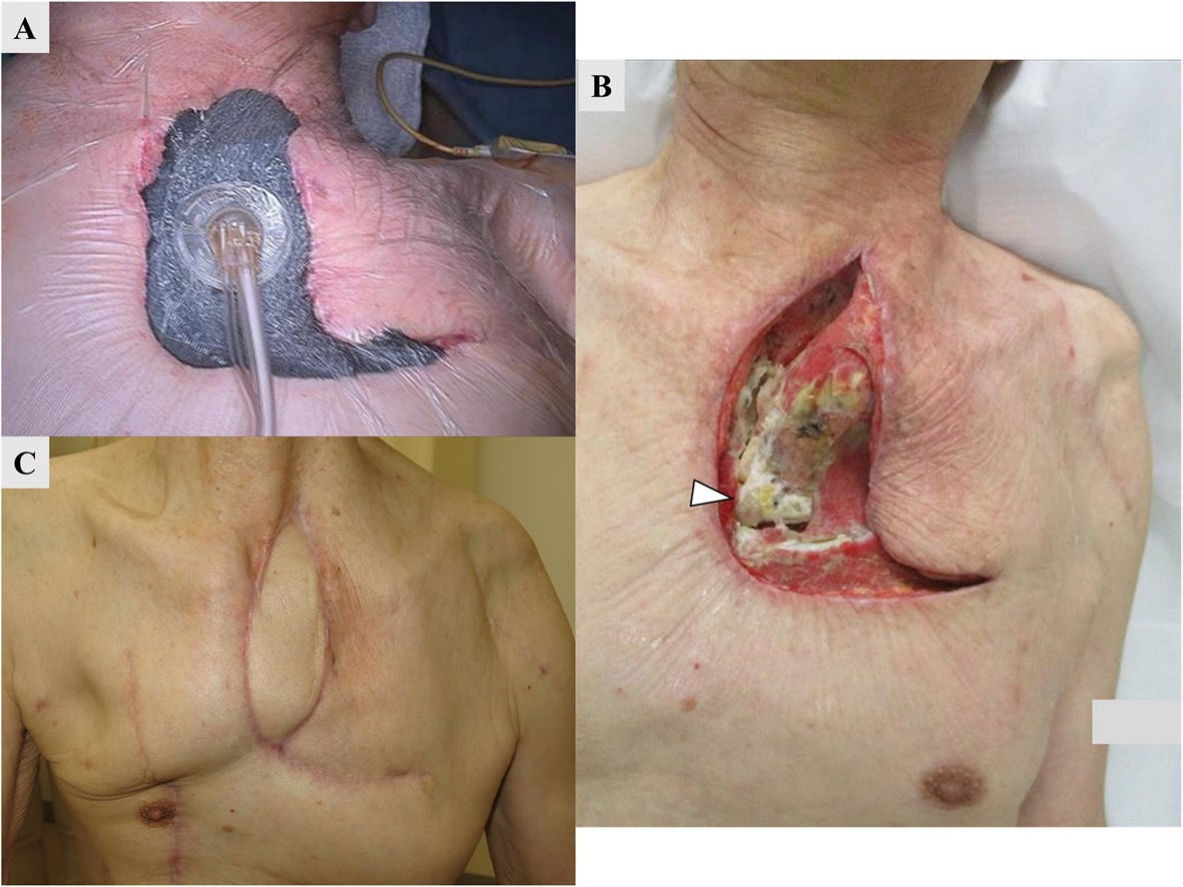
Negative pressure wound therapy was performed for 17 days (**A**). After the negative pressure wound therapy (**B**), the infection was controlled, but the manubrium of the sternum developed a sequestrum (white arrowhead). The wound healed after a sequestrectomy of the manubrium and chest wall reconstruction using a right pectoralis major myocutaneous flap (**C**)

The patient was extubated on day 1 but required non-invasive positive pressure ventilation for type II respiratory failure until day 5. On day 9, mediastinitis developed, likely due to a sternal wire infection, as anticipated preoperatively. Debridement, sternal wire removal, antibiotic therapy, and negative pressure wound therapy (NPWT) were initiated (Fig. 6A). Methicillin-resistant Staphylococcus epidermidis was cultured from the surgical site, and Citrobacter koseri was identified in blood cultures. Consequently, meropenem at 3 g/day and vancomycin at 2 g/day was intravenously administrated. After 17 days of antibiotic therapy and NPWT, the infection subsided (Fig. 6B), and the patient underwent a sequestrectomy of the affected manubrium with a chest wall reconstruction using a pedicled right pectoralis major myocutaneous flap (Fig. 6C). The antibiotic regimen was transitioned to oral linezolid at a dosage of 1.2 g, which was continued for a duration of 2 months postoperatively. Wound healing was satisfactory, with no recurrence of the infection at 12 months after the reconstructive surgery. The patient had a mild limitation of the left upper limb abduction but no impairment of the activities of daily living.

## Discussion

Robot-assisted thoracic surgery (RATS) is becoming increasingly feasible for anterior mediastinal tumors, offering an alternative to a conventional median sternotomy and video-assisted thoracic surgery (VATS) [1, 2]. RATS provides less invasiveness through the secure fixation of ports to the thoracic cage, better maneuverability based on multi-articulated arms, and more excellent field of view due to a high-resolution three-dimensional camera than conventional approaches [3, 4]. Despite these advantages, open surgery such as a median sternotomy remains the mainstay for cases of vascular invasion or multi-organ resections [5, 6]. The TMA, initially proposed for apical chest tumors, allows access to the subclavicular region while sparing the clavicle and muscular attachments [7]. However, a TMA alone is not suitable for cases requiring an anatomical lung resection, necessitating an additional thoracotomy [7, 8]. In such cases, an extended cervicosternothoracotomy [9] and hemi-clamshell technique [10] provide access for a thymectomy and lung resection but cause substantial disruption of the osseous thoracic cage, leading to increased morbidity [7, 11, 12]. Thoracoscopic surgery in the lateral decubitus position remains the preferred approach for anatomical lung resections, offering a better surgical view and reduced invasiveness [13, 14].

In this case, we determined that initiating the procedure via RATS is significantly superior. The reasons are as follows. The suspected invasion extended to the distal portion of the left brachiocephalic vein, making it essential to evaluate resectability with clearer image quality and enhanced maneuverability. While conventional VATS offers only a linear approach, RATS provides superior maneuverability through its multi-articulated arms. Additionally, the high-resolution 3D imaging system of RATS offers a better operative field compared to conventional VATS, potentially enhancing surgical precision and safety. Based on a comprehensive consideration of these factors, we concluded that RATS is the most appropriate choice in this case.

However, the following challenges were anticipated in performing RATS from the right side in this case: (1) concern about tumor invasion into the left brachiocephalic vein and/or left lung, (2) difficulty in identifying the left vagus and recurrent laryngeal nerves within the thoracic cavity due to the large tumor size and poor dorsal visibility.

**Fig. 7:**
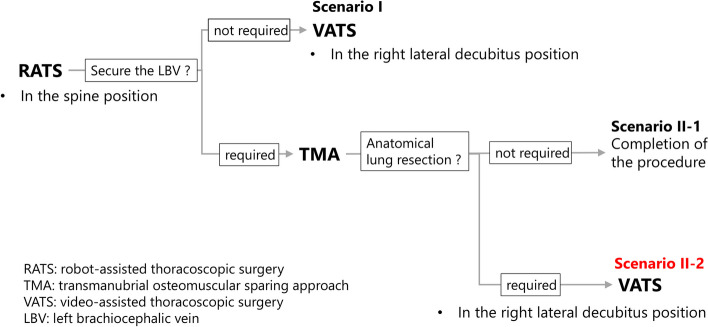
Flowchart of the preoperative planning. If securing the left brachiocephalic vein is unnecessary, proceed with Scenario I; if necessary, proceed with Scenario II. Furthermore, if an anatomical lung resection is not required, proceed with Scenario II-1; if required, proceed with Scenario II-2. In the actual surgery, Scenario II-2 was followed

We preoperatively devised the following scenarios to address these issues (Fig. 7): Scenario I) If a tumor dissection from the left brachiocephalic vein was easily achievable, a robotic thymothymectomy from the right side in the supine position would be performed, progressing as far as possible into the left hemithorax. Given the large tumor size, a subsequent mini-thoracotomy in the lateral decubitus position would be necessary anyway to retrieve the specimen. Scenario II) If the dissection proved challenging, open surgery via a TMA would be added to safely secure the left brachiocephalic vein, avoiding the need for a total sternotomy. The TMA also allows for the identification of the vagus nerve under direct vision, minimizing the risk of injury to both the vagus and recurrent laryngeal nerves. If a combined anatomical lung resection was not required, the procedure would be completed by retrieving the specimen through the same incision (Scenario II-1). However, if a combined anatomical lung resection was required, to avoid an additional sternotomy or excessive thoracotomy, we planned to perform the resection via thoracoscopic surgery with the patient in the right lateral decubitus position (Scenario II-2). Although robotic surgery was feasible, we chose conventional thoracoscopic surgery to maximize the benefits of a mini-thoracotomy for specimen retrieval, as in Scenario I.

In the present case, the TMA was performed according to Scenario II due to a firm adhesion to the left brachiocephalic vein. Additionally, because a combined left upper segmentectomy and extensive dorsal adhesiolysis were necessary, the patient was transitioned into the right lateral decubitus position, and thoracoscopic surgery was performed following Scenario II-2.

Sternal osteomyelitis and mediastinitis after the median sternotomy are serious complications, with an incidence of 1 to 4% [15–17] and a reported mortality rate of 14 to 47% [18, 19]. The treatment strategy typically involves debridement of the necrotic sternum, followed by reconstruction of the defect [20]. Wound closure requires an autologous tissue reconstruction, such as a musculocutaneous flap. Had a total sternotomy been performed in the present case, a larger area would likely have been affected, necessitating more extensive debridement and a larger graft volume, complicating the reconstruction [21]. Additionally, the NPWT may have accelerated wound healing and contributed to successful reconstruction by enhancing microvascular blood flow, promoting granulation tissue formation, and reducing the bacterial load [22, 23].

The limitation of the multidisciplinary approach in this case lies in the complexity of the procedure and the resultant prolonged operative time. The conversion from robotic surgery to conventional thoracoscopic surgery, along with the repositioning from supine to lateral decubitus, contributed to the extended duration. The prolonged operative time itself may have adversely affected the postoperative surgical site infection risk. However, the combination of the procedures minimized the osseous chest wall destruction, which helped to reduce any postoperative respiratory failure and eased the burden of subsequent chest wall reconstruction. This case was at high risk for a surgical site infection due to immunosuppression. By avoiding a total sternotomy, the extent of the mediastinitis was localized, allowing for a limited sequestrectomy with a favorable outcome. Meticulous preoperative planning also facilitated intraoperative decision-making and yielded a successful outcome. These results support the validity of our strategy.

## Conclusions

Despite the complexity and prolonged operative time, a multidisciplinary approach combining robotic and conventional surgical techniques offers a feasible option for managing an invasive thymoma, particularly in immunocompromised patients. Preoperative surgical planning is essential for guiding intraoperative decision-making and ensuring optimal outcomes.

## Data Availability

Data sharing is not applicable to this article as no datasets were generated or analyzed during the current study.

## Abbreviations

CT: Chest computed tomography
FPS: Fasciitis panniculitis syndrome
TMA: Transmanubrial osteomuscular sparing approach
NPWT: Negative pressure wound therapy
